# Classroom-Level and Individual-Level Prosociality and Help-Seeking Behaviors Among Adolescents

**DOI:** 10.1001/jamanetworkopen.2025.10319

**Published:** 2025-05-15

**Authors:** Ryo Morishima, Satoshi Usami, Akiko Kanehara, Naohiro Okada, Haruko Noguchi, Sho Yagishita, Masato Fukuda, Kiyoto Kasai

**Affiliations:** 1 Department of Neuropsychiatry, Graduate School of Medicine, The University of Tokyo, Tokyo, Japan; 2 Department of Psychology, Faculty of Health and Medical Science, Teikyo Heisei University, Tokyo, Japan; 3 Waseda Institute of Social and Human Capital Studies, Tokyo, Japan; 4 Division of Educational Psychology, Graduate School of Education, The University of Tokyo, Tokyo, Japan; 5 International Research Center for Neurointelligence, The University of Tokyo Institutes for Advanced Study, Tokyo, Japan; 6 Faculty of Political Science and Economics, Waseda University, Tokyo, Japan.; 7 Department of Structural Physiology, Graduate School of Medicine, The University of Tokyo, Tokyo, Japan; 8 Department of Psychiatry and Neuroscience, Graduate School of Medicine, Gunma University, Gunma, Japan

## Abstract

**Importance:**

Although individual prosociality, defined as voluntary behavior intended to benefit another, is known to promote help-seeking behaviors among adolescents, it remains unclear whether a prosocial community, such as the classroom, also facilitates these behaviors.

**Objective:**

To investigate the associations of classroom-level and individual-level prosociality with help-seeking behaviors, considering age and gender, and to examine moderation effects of being bullied.

**Design, Setting, and Participants:**

In this cross-sectional study, annual surveys were conducted via questionnaire among students in Japanese junior and senior high schools from 2020 to 2023. The first wave was administered from October 1 to November 7, 2020, followed by the second wave from June 4 to July 13, 2021, the third wave from June 17 to July 19, 2022, and the fourth wave from June 19 to July 28, 2023.

**Exposure:**

Classroom-level prosociality was defined as the mean prosociality of all students within a classroom, while individual-level prosociality was measured as the deviation from this classroom mean. Prosociality was assessed using the prosocial subscale of the self-reported Strengths and Difficulties Questionnaire.

**Main Outcomes and Measures:**

Generalized mixed-effects models estimated the associations of classroom-level and individual-level prosociality with help-seeking behaviors (including poor help-seeking and seeking help from friends, family members, homeroom teachers, and physicians) while considering subgroups based on grades and gender, as well as the moderation effect of being bullied. Help-seeking behaviors were evaluated by a question about seeking help for one’s mental health problems (“Are you currently consulting anyone to discuss your psychological stress or mental health problems?”), with multiple options. The robustness of the main findings were tested by models stratified by survey year.

**Results:**

The analysis included 21 845 participants (mean [SD] school grade, 10.4 [1.2]; 16.6% junior high school students and 83.4% senior high school students; 53.3% boys). Classroom-level prosociality was associated with a higher rate of help-seeking from friends among senior high school students (odds ratio [OR], 1.26, 95% CI, 1.17-1.35), which confirmed the robustness. No moderation effect of being bullied was found in this association. Higher individual-level prosociality was associated with increased help-seeking from various sources across nearly all models and subgroups, regardless of being bullied (friends: OR, 1.15 [95% CI, 1.13-1.17]; *P* < .001; family members: OR, 1.09 [95% CI, 1.07-1.11]; *P* < .001; homeroom teachers: OR, 1.15 [95% CI, 1.10-1.19]; *P* < .001; school nurses: OR, 1.19 [95% CI, 1.12-1.28]; *P* < .001; and physicians: OR, 1.14 [95% CI, 1.07-1.20]; *P* < .001).

**Conclusions and Relevance:**

This cross-sectional study of 21 845 students found that both classroom-level and individual-level prosociality were associated with higher rates of help-seeking from peers among senior high school students, while accounting for individual characteristics. Enhancing classroom prosociality could foster help-seeking behaviors through reciprocal peer support, benefiting even those who experienced bullying.

## Key Points

**Question:**

Can the association between prosociality and help-seeking behaviors among students be partially explained by classroom effects?

**Findings:**

In this cross-sectional study of 21 845 students, annual surveys conducted via questionnaire in Japanese junior and senior high schools revealed that both classroom-level and individual-level prosociality were associated with higher rates of help-seeking from friends among senior high school students. This association remained significant after controlling for individual characteristics, such as general psychopathology, and despite experiences of being bullied.

**Meaning:**

This finding suggests that a prosocial classroom climate in senior high school may facilitate help-seeking from peers, regardless of individual student characteristics.

## Teaser

This cross-sectional survey study investigates the associations of classroom-level and individual-level prosociality with help-seeking behaviors among adolescents in Japan and examines the moderation effects of being bullied.

## Introduction

Adolescence is a critical period for the onset of various mental disorders,^1,2^ highlighting the importance of fostering help-seeking behaviors. The proportion of adolescents who refrain from seeking help cannot be ignored.^3,4,5^ A recent report indicated that approximately 1 in 5 adolescents do not seek help from either informal (eg, friends, family) or formal sources (eg, counselors, physicians), despite having support needs.^6^ This group is considered to be at high risk for suicidal ideation and self-harm.^7,8^ Furthermore, adolescents with severe mental health problems are even less likely to seek help.^9,10,11^ It is crucial to explore the determinants that promote help-seeking behaviors, irrespective of individual characteristics, to create supportive environments that enhance adolescent mental health.

Prosociality, defined as voluntary behavior intended to benefit another,^12^ not only contributes to personal well-being but also engenders positive behavioral and psychological changes within one’s social environment.^13,14,15,16^ Previous studies have indicated that adolescents with higher level of prosociality are more likely to seek help.^10,17,18,19^ Two potential pathways may underlie this association. First, individuals perceiving greater support from their social environment are more inclined to seek help,^10,20^ suggesting that belonging to a prosocial community (eg, a classroom) facilitates help-seeking behaviors among its members. Second, those who frequently engaged in helping behaviors may witness the positive outcomes of receiving help, thereby reducing their own barriers to seeking help in the future. Although stigma and negative attitudes toward help-seeking are major barriers,^9,20,21^ prosocial experiences may help mitigate personal resistance to engaging in help-seeking behaviors. Thus, both classroom-level and individual-level prosociality may be associated with an increased likelihood of help-seeking behaviors among adolescents. However, previous studies have not clearly distinguished between these 2 levels, making the 2 potential pathways unclear. Moreover, subgroup analyses by age and gender are recommended, as older age and being female have been associated with higher rates of help-seeking behaviors and greater likelihood of engaging in prosocial behavior.^6,22,23,24,25,26,27^ Providing empirical evidence of classroom-level prosociality as a determinant of enhancing help-seeking behaviors and its association with subgroup differences could reveal the advantage of an approach targeting prosociality for all classroom members, regardless of individual prosociality, while considering adolescents’ developmental stage and gender.

However, the associations of prosociality with help-seeking behavior may be moderated by being bullied. Adolescents who experience bullying and do not seek help are at increased risk of psychological distress.^28^ Fear of retaliation and a sense of self-imposed responsibility to manage the situation can hinder help-seeking.^29,30,31^ Although negative perceptions of available support sources represent an additional barrier to help-seeking behaviors,^9,21,32^ being bullied may impede feelings of safety in the community.^33,34,35,36^ Even within a highly prosocial classroom, adolescents who experience bullying may still be reluctant to seek help.

The main focus in the present study was to expand our understanding of whether the association between prosociality and help-seeking behaviors among students can be partially explained by classroom effects. Therefore, this study aimed to (1) examine the association of classroom-level, as well as individual-level, prosociality with help-seeking behaviors among adolescents, (2) examine the subgroup differences in the associations between classroom-level prosociality and help-seeking behaviors, and (3) investigate the moderation effect of being bullied. We hypothesized that greater classroom-level prosociality and individual prosociality would each be associated with higher rates of help-seeking behavior and that the associations of classroom-level prosociality would be more pronounced among older compared with younger adolescents and among girls compared with boys. Furthermore, we anticipated that being bullied would be adversely associated with the link between classroom-level prosociality and help-seeking behaviors.

## Methods

### Study Design and Setting

The School Adolescent Behavior and Care (S-ABC) survey is an annual, multiwave cross-sectional survey conducted in the Saitama prefecture of Japan. Detailed descriptions of the survey are available in previous studies^6,37^ and in the eMethods in Supplement 1. This study was approved by the Ethics Committee of the Faculty of Medicine at the University of Tokyo and the Ethics Committee of Teikyo Heisei University. Participants’ willingness to participate was verified by obtaining their written informed consent. The anonymous survey commenced with the first wave conducted across 21 schools from October 1 to November 7, 2020. Subsequent waves were administered as follows: the second wave in 28 schools (June 4-July 13, 2021), the third wave in 26 schools (June 17-July 19, 2022), and the fourth wave in 25 schools (June 19-July 28, 2023). A flowchart of participant selection can be found in the eFigure in Supplement 1. Throughout the 4 waves, the response rate was consistently high, ranging from 88.9% to 90.8%. In total, this study included 748 classrooms with enrolled students (167 classrooms in 2020, 218 classrooms in 2021, 193 classrooms in 2022, and 170 classrooms in 2023). This study adhered to the Strengthening the Reporting of Observational Studies in Epidemiology (STROBE) reporting guidelines.

### Measurements

Prosociality was assessed using the prosocial subscale of the self-reported Strengths and Difficulties Questionnaire (SDQ).^38,39^ This subscale comprises 5 items, with responses scored as 0 (“not true”), 1 (“somewhat true”), and 2 (“certainly true”). The total subscale score ranges from 0 to 10, with higher scores indicating higher prosocial tendency. We calculated both classroom-level and individual-level prosociality scores.^40^ The classroom-level score reflects the mean prosociality level of students within a classroom, while the individual score represents the deviation from the classroom mean (ie, centering within clusters).

Help-seeking behaviors were evaluated using the question “Are you currently consulting anyone to discuss your psychological stress or mental health problems?” Response options included “No, I do not need to consult because I have no psychological stress or mental health problems”; “No, I am not currently consulting anyone despite having some psychological stress or mental health problems”; and “Yes, I am currently in discussion about my psychological stress or mental health problems.” Adolescents who selected the second response option were categorized as having poor help-seeking.^6,7,41^ For those selecting the third option, all sources for consultation were identified from a list of potential sources for help-seeking (friends, family members, homeroom teachers, school nurses, school counselors, counselors outside of school, physicians, and others) (Table 1).

**Table 1.** Descriptive Statistics of the Study Participants by Survey Year

Characteristic	Participants, No. (%)				
	Total (N = 21 845)	2020 (n = 5000)	2021 (n = 6062)	2022 (n = 5659)	2023 (n = 5124)
Grade					
Junior high school	3622 (16.6)	780 (15.6)	994 (16.4)	1104 (19.5)	744 (14.5)
Senior high school	18 223 (83.4)	4220 (84.4)	5068 (83.6)	4555 (80.5)	4380 (85.5)
Gender					
Boy	11 638 (53.3)	2739 (54.8)	2965 (48.9)	3127 (55.3)	2807 (54.8)
Girl	9952 (45.6)	2214 (44.3)	3031 (50.0)	2452 (43.3)	2255 (44.0)
Other^a^	100 (0.5)	24 (0.5)	24 (0.4)	24 (0.4)	28 (0.6)
General psychopathology, mean (SD) SDQ total difficulties score^b^	12.1 (5.3)	11.9 (5.2)	12.4 (5.4)	12.0 (5.2)	12.1 (5.5)
Being bullied	3544 (16.2)	869 (17.4)	987 (16.3)	883 (15.6)	805 (15.7)
Prosociality, mean (SD) SDQ prosocial subscale score^c^	6.1 (2.2)	6.1 (2.2)	6.2 (2.2)	6.1 (2.2)	6.1 (2.2)
Classroom-level prosociality, mean (SD)	6.1 (0.6)	6.1 (0.6)	6.2 (0.6)	6.1 (0.6)	6.1 (0.5)
Individual-level prosociality, mean (SD)	0.00 (2.1)	0.0 (2.1)	0.0 (2.1)	0.0 (2.1)	0.0 (2.2)
Poor help-seeking	4087 (18.7)	815 (16.3)	1226 (20.2)	1097 (19.4)	949 (18.5)
Help-seeking from					
Friends	5827 (26.7)	1440 (28.8)	1586 (26.2)	1549 (27.4)	1252 (24.4)
Family members	5197 (23.8)	1223 (24.5)	1441 (23.8)	1333 (23.6)	1200 (23.4)
Homeroom teacher	637 (2.9)	150 (3.0)	161 (2.7)	163 (2.9)	163 (3.2)
School nurses	222 (1.0)	46 (0.9)	59 (1.0)	62 (1.1)	55 (1.1)
School counselors	155 (0.7)	27 (0.5)	57 (0.9)	35 (0.6)	36 (0.7)
Counselors outside of school	113 (0.5)	24 (0.5)	39 (0.6)	26 (0.5)	24 (0.5)
Physicians	300 (1.4)	67 (1.3)	78 (1.3)	82 (1.5)	73 (1.4)
Others	368 (1.7)	84 (1.7)	93 (1.5)	89 (1.6)	102 (2.0)

Abbreviation: SDQ, Strengths and Difficulties Questionnaire.

^a^ Respondents could self-select the other gender category.

^b^ Responses were summed across 19 items assessing emotional and behavioral problems to yield a total score ranging from 0 to 38, with higher scores indicating a higher state of emotional and behavioral problems.

^c^ Responses were summed across 5 items to yield a total score ranging from 0 to 10, with higher scores indicating higher prosocial tendency.

Student experience of bullying was assessed using an item from the peer problems subscale of the SDQ: “Other children or young people pick on me or bully me,” rated on a 3-point Likert scale from 0 to 2. Scores of 1 or 2 indicated being bullied, while a score of 0 indicated not being bullied.

Our analyses included grades (junior or senior high school), gender (boy, girl, or other), and general psychopathology. We categorized grades following the Japanese education system, with high schools divided into junior high school (ages 12-15 years correspond to grades 7-9) and senior high school (ages 15-18 years correspond to grades 10-12). General psychopathology was assessed using the total difficulties scores of the SDQ.^38,39^ One item on being bullied was excluded from the total score, thus summing responses across 19 items assessing emotional and behavioral problems to yield a total score ranging from 0 to 38, with higher scores indicating a higher state of emotional and behavioral problems.

### Statistical Analysis

To explore the associations of classroom-level and individual-level prosociality with help-seeking behaviors, we used generalized mixed-effects models following binomial distribution, adjusting for grade, gender, and general psychopathology. Each model included the classroom variable as a random intercept. Models were compared to determine the necessity of adding a random slope using the Akaike information criterion (AIC) and the bayesian information criterion (BIC). The model comparisons included (1) random intercept only, (2) random intercept with a random slope for the classroom-level effect of prosociality, (3) random intercept with a random slope for the individual-level effect of prosociality, and (4) random intercept with random slopes for both classroom-level and individual-level effects of prosociality. When these indices preferred different models, we selected a model with the lowest BIC value because the BIC outperformed the AIC in cases with large sample sizes.^42,43^

Subgroup analyses were conducted based on grades and gender. After the assessment of interaction effects between classroom-level and/or individual-level prosociality and grades and gender on help-seeking behaviors, we compared models with random intercepts to determine if adding a random slope improved model fit, using the AIC and BIC in the same manner as for the total sample. We further examined the interaction effect of grade level (grades 7-9 in junior high school and grades 10-12 in senior high school) and classroom-level prosociality on help-seeking behaviors in the modes indicating significant results of classroom-level prosociality in each grade subgroup. Gender subgroup analyses were performed only for boys and girls, as the sample size for the “other” gender group was small (100 of 21 845 [0.5%]) (Table 1).

To examine the moderation effect of being bullied, we tested interaction effects between classroom-level and/or individual-level status of prosociality and being bullied in the modes, showing significant results among the total sample and each subgroup. When significant interaction terms were identified, models stratified by experience of bullying were also conducted.

Sensitivity analyses were conducted to assess the robustness of the classroom-level effect of prosociality, using stratified models from survey years (2020, 2021, 2022, and 2023).

All models estimated odds ratios (ORs) and 95% CIs. Missing data were addressed using multiple imputations, generating 20 imputed datasets. The analyses were performed using R, version 4.1.2 (R Project for Statistical Computing), with the lme4 and mice packages for generalized mixed-effects models and multiple imputation. *P* values for the estimations of ORs and 95% CIs were from 2-sided tests and results were deemed statistically significant at *P* < .05. The significance level was set to α = .10 for interaction terms when considering effect measure modification.

## Results

The analysis included 21 845 participants (mean [SD] school grade, 10.4 [1.2]; 16.6% junior high school students and 83.4% senior high school students; 53.3% boys and 45.6% girls). Table 1 presents descriptive statistics for the participants. The missing observations for each variable are summarized in eTable 1 in Supplement 1.

Table 2 details the pooled results from 20 models generated by multiple imputations with the lowest mean AIC and BIC values. Additional information is provided in eTable 2 in Supplement 1. Higher classroom-level prosociality and individual-level prosociality were associated with lower rates of poor help-seeking (classroom-level prosociality: OR, 0.89, 95% CI; 0.83-0.96; individual-level prosociality: OR, 0.97; 95% CI, 0.95-0.98) and higher rates of help-seeking from friends (classroom-level prosociality: OR, 1.23; 95% CI, 1.15-1.32; individual-level prosociality: OR, 1.15; 95% CI, 1.13-1.17) (Table 2). Higher individual-level prosociality was associated with help-seeking from friends (OR, 1.15 [95% CI, 1.13-1.17]; *P* < .001), family members (OR, 1.09 [95% CI, 1.07-1.11]; *P* < .001), homeroom teachers (OR, 1.15 [95% CI, 1.10-1.19]; *P* < .001), school nurses (OR, 1.19 [95% CI, 1.12-1.28]; *P* < .001), and physicians (OR, 1.14 [95% CI, 1.07-1.20]; *P* < .001).

**Table 2.** Results of Generalized Mixed-Effects Models of Association of Classroom-Level and Individual-Level Prosociality With Help-Seeking Behaviors

Characteristic	OR (95% CI)^a^	*P* value
**Poor help-seeking**		
Classroom-level prosociality	0.89 (0.83-0.96)	.002
Individual-level prosociality	0.97 (0.95-0.98)	<.001
**Help-seeking from**		
Friends		
Classroom-level prosociality	1.23 (1.15-1.32)	<.001
Individual-level prosociality	1.15 (1.13-1.17)	<.001
Family members		
Classroom-level prosociality	1.03 (0.97-1.11)	.34
Individual-level prosociality	1.09 (1.07-1.11)	<.001
Homeroom teachers		
Classroom-level prosociality	0.91 (0.76-1.09)	.29
Individual-level prosociality	1.15 (1.10-1.19)	<.001
School nurses		
Classroom-level prosociality	1.02 (0.78-1.33)	.89
Individual-level prosociality	1.19 (1.12-1.28)	<.001
School counselors		
Classroom-level prosociality	1.17 (0.84-1.62)	.36
Individual-level prosociality	1.00 (0.93-1.08)	.92
Counselors outside of school		
Classroom-level prosociality	0.97 (0.68-1.37)	.84
Individual-level prosociality	1.01 (0.92-1.10)	.86
Physicians		
Classroom-level prosociality	1.18 (0.94-1.49)	.15
Individual-level prosociality	1.14 (1.07-1.20)	<.001
Others		
Classroom-level prosociality	0.99 (0.82-1.21)	.95
Individual-level prosociality	1.03 (0.98-1.08)	.23

Abbreviation: OR, odds ratio.

^a^ All models adjusted for grade, gender, and general psychopathology.

For the subgroup analyses, we conducted subsequent stratified analyses when the interaction effects of prosociality with grades and gender were significant (eTable 3 in Supplement 1). Classroom-level prosociality was significantly associated with help-seeking from friends among senior high school students (OR, 1.26; 95% CI, 1.17-1.35) and help-seeking from physicians among junior high school students in each best-fitting model (OR, 2.73; 95% CI, 1.40-5.35; Table 3; eTable 4 in Supplement 1). Unlike the total sample results, individual-level prosociality was significantly associated with help-seeking from counselors outside of school among junior high school students and from others among girls. We found no interaction effect between grade level and classroom-level and individual-level prosociality on help-seeking from physicians among junior high school students (classroom by grades 7-9: OR, 0.81 [95% CI, 0.10-6.68]; *P* = .85; individual by grades 7-9: OR, 0.81 [95% CI, 0.56-1.17]; *P* = .26), and friends among senior high school students (classroom by grades 10-12: OR, 0.91 [95% CI, 0.77-1.07]; *P* = .26; individual by grades 10-12: OR, 1.02 [95% CI, 0.98-1.07]; *P* = .31).

**Table 3.** Results of Generalized Mixed-Effects Models of Association of Classroom-Level and Individual-Level Prosociality With Help-Seeking Behaviors in Each Subgroup

Characteristic	OR (95% CI)^a^	*P* value
**Junior high school**		
Help-seeking from		
Friends		
Classroom-level prosociality	1.10 (0.91-1.33)	.33
Individual-level prosociality	1.11 (1.06-1.15)	<.001
School nurses		
Classroom-level prosociality	1.87 (0.95-3.67)	.07
Individual-level prosociality	1.26 (1.08-1.49)	.005
Counselors outside of school		
Classroom-level prosociality	1.51 (0.53-4.29)	.44
Individual-level prosociality	1.28 (1.02-1.60)	.03
Physicians		
Classroom-level prosociality	2.73 (1.40-5.35)	.003
Individual-level prosociality	1.08 (0.92-1.26)	.33
Others		
Classroom-level prosociality	0.72 (0.46-1.12)	.14
Individual-level prosociality	0.98 (0.89-1.08)	.67
**Senior high school**		
Help-seeking from		
Friends		
Classroom-level prosociality	1.26 (1.17-1.35)	<.001
Individual-level prosociality	1.16 (1.14-1.18)	<.001
School nurses		
Classroom-level prosociality	0.92 (0.69-1.22)	.55
Individual-level prosociality	1.18 (1.10-1.27)	<.001
Counselors outside of school		
Classroom-level prosociality	0.88 (0.60-1.28)	.50
Individual-level prosociality	0.96 (0.88-1.06)	.42
Physicians		
Classroom-level prosociality	1.07 (0.84-1.36)	.58
Individual-level prosociality	1.14 (1.08-1.22)	<.001
Others		
Classroom-level prosociality	1.09 (0.87-1.37)	.43
Individual-level prosociality	1.05 (0.99-1.11)	.10
**Boys**		
Help-seeking from		
Family members		
Classroom-level prosociality	0.99 (0.90-1.09)	.82
Individual-level prosociality	1.10 (1.07-1.13)	<.001
Others		
Classroom-level prosociality	1.09 (0.79-1.50)	.61
Individual-level prosociality	0.90 (0.77-1.05)	.18
**Girls**		
Help-seeking from		
Family members		
Classroom-level prosociality	1.06 (0.98-1.16)	.17
Individual-level prosociality	1.08 (1.06-1.11)	<.001
Others		
Classroom-level prosociality	0.96 (0.72-1.27)	.76
Individual-level prosociality	1.10 (1.02-1.17)	.01

Abbreviation: OR, odds ratio.

^a^ All models adjusted for gender and general psychopathology.

After examining interaction effects (eTable 6 in Supplement 1), stratified results by whether students had been bullied indicated no moderation effect on classroom-level prosociality in the total sample (Table 4). Higher individual-level prosociality was associated with increased help-seeking from homeroom teachers among students who were not bullied but not in students who were. In all subgroups, the results showed no moderation effect of being bullied on the association between classroom-level prosociality and help-seeking behaviors (eTable 6 in Supplement 1; Table 4).

**Table 4.** Results of Generalized Mixed-Effects Models of Association of Classroom- and Individual-Level Prosociality With Help-Seeking Behaviors According to Whether Students Were Bullied

Characteristic	Students who were bullied		Students who were not bullied	
	OR (95%CI)	*P* value	OR (95%CI)	*P* value
**Total sample^a^**				
Help-seeking from				
Family members				
Classroom-level prosociality	0.90 (0.77-1.04)	.16	1.07 (1.00-1.16)	.06
Individual-level prosociality	1.11 (1.06-1.15)	<.001	1.09 (1.07-1.11)	<.001
Homeroom teacher				
Classroom-level prosociality	0.80 (0.57-1.11)	.18	0.96 (0.77-1.19)	.70
Individual-level prosociality	1.04 (0.96-1.12)	.37	1.19 (1.13-1.25)	<.001
**Junior high school^b^**				
Help-seeking from				
Friends				
Classroom-level prosociality	1.17 (0.83-1.63)	.37	1.09 (0.86-1.36)	.48
Individual-level prosociality	1.18 (1.09-1.28)	<.001	1.08 (1.03-1.13)	.001
**Boys^c^**				
Help-seeking from				
Family members				
Classroom-level prosociality	0.85 (0.70-1.03)	.10	1.04 (0.93-1.17)	.49
Individual-level prosociality	1.11 (1.05-1.16)	<.001	1.10 (1.07-1.13)	<.001

Abbreviation: OR, odds ratio.

^a^ Model adjusted for grade, gender, and general psychopathology.

^b^ Model adjusted for gender and general psychopathology.

^c^ Model adjusted for grade and general psychopathology.

In the sensitivity analyses, models stratified by survey year were conducted for 3 models to verify the robustness of the significant classroom-level effects. Across 4 waves, we confirmed the robust association between classroom-level prosociality and help-seeking from friends among senior high school students (Figure). We did not observe a consistent association between classroom-level prosociality and poor help-seeking among the total sample and help-seeking from physicians among junior high school students (eTable 7 in Supplement 1). Among the total sample, classroom-level prosociality was significantly associated with poor help-seeking only in 2023 (OR, 0.80 [95% CI, 0.70-0.94]; *P* = .009). Among junior high school students, classroom-level prosociality was associated with help-seeking from physician only in 2020 (OR, 7.16 [95% CI, 2.18-23.52]; *P* = .001).

**Figure.**
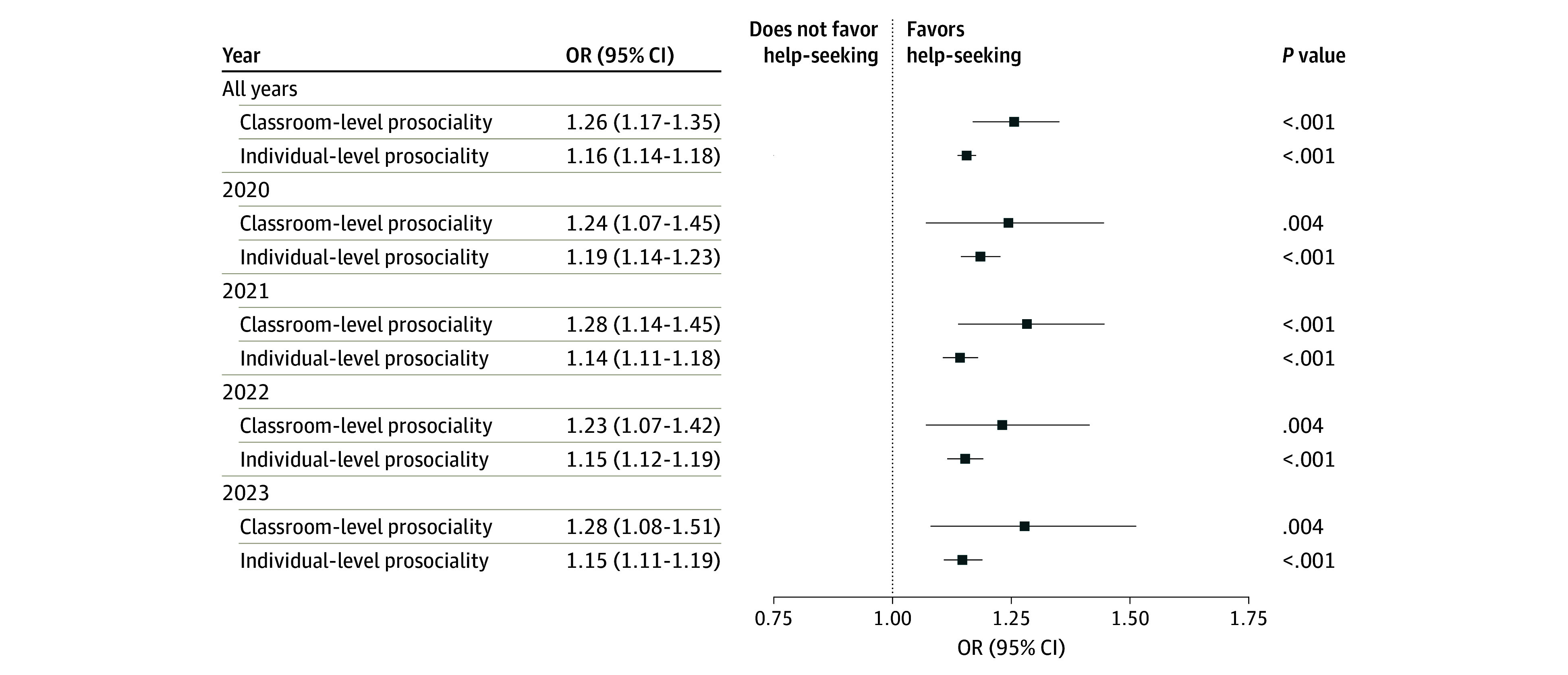
Generalized Mixed-Effects Models of the Association of Classroom-Level and Individual-Level Prosociality With Help-Seeking From Friends Among Senior High School Students The models adjusted for gender and general psychopathology with the classroom variable as a random intercept.

## Discussion

To our knowledge, this study represents the first investigation into the association between classroom-level prosociality and help-seeking behaviors while accounting for individual characteristics, such as individual-level prosociality and general psychopathology. Our findings suggest that this association remains significant for help-seeking behaviors from friends among senior high school students across gender, experience of bullying, and survey years, supporting existing literature that associates perceived social support with help-seeking behaviors or intentions.^10,20^ However, individual mental health conditions can be associated with perceived social support or help-seeking behaviors or intentions.^9,10,11,44^ Individuals with a greater need for support might face challenges in leveraging social support effectively. Our results suggest that among senior high school students, a prosocial classroom environment may encourage help-seeking behaviors for all members, including those in greater need of mental health support. Adolescents perceive peers as a lower-risk source of help-seeking,^30^ and those with trusted classmates may be more inclined to seek help from them during late adolescence.

In the sensitivity analyses, classroom-level prosociality was associated with help-seeking from physicians in 2020 among junior high school students and was associated with poor help-seeking in 2023 among the total sample. Psychological response during the COVID-19 pandemic might have changed the association of classroom-level prosociality with help-seeking behaviors. In the early phase of the pandemic, physical distancing may have reduced adolescents’ opportunities to engage in social contact^45^ and may have led to situations that inhibited their help-seeking behaviors. Given that the medical systems in Japan allow pediatricians to treat students until approximately 15 years of age, when junior high school students (13-15 years) or their family members were infected with COVID-19, the medical staff may have assessed them for psychosocial challenges to avoid the stigma associated with COVID-19 infection.^46^ These assessments may have been associated with the difference in our findings by developmental stage and survey year.

Our findings have key implications. Enhancing classroom prosociality may promote help-seeking behaviors among senior high school students, irrespective of individual prosociality levels or general psychopathology. As universal prevention approaches in school settings,^47^ implementing educational programs, group-based psychological interventions, and social initiatives that enhance classroom-level prosociality could encourage help-seeking behaviors.^48,49^ Our findings support the robustness of this association for even the COVID-19 pandemic period, when the number of adolescents who sought help decreased.^6^ In addition, because stronger peer relationships and a greater sense of belonging in high school have been shown to be associated with higher relational health in adulthood regardless of adverse childhood experiences,^50^ a prosocial classroom environment may be associated not only with current help-seeking behaviors but also with future relational health. Furthermore, research examining the applicability of this finding to other mental health outcomes is needed, as it might be associated with the group dynamics that lead to positive behavioral and psychological changes, such as the normalization of mental disorders or the inhibition of antisocial behavior.^15,51^

Our study provides novel insights into the association between individual-level prosociality and help-seeking from friends, family members, homeroom teachers, and school nurses, controlling for classroom-level prosociality—a distinction not previously addressed.^10,17,18,19^ Higher individual-level prosociality may reduce stigma and negative attitudes associated with help-seeking, as individuals who help others are more likely to seek help themselves.^9,20,21^ In the group of students that experienced bullying, help-seeking from homeroom teachers was not significant, which could reflect a diminished sense of safety or trust in teachers among these students,^33,34,35,36^ despite teachers’ crucial role in fostering classroom safety. Among junior high school students, individual-level prosociality was associated with increased help-seeking from counselors outside of school. Younger adolescents, compared with older adolescents, typically report higher family cohesion^52^ and are more likely to seek help from parents, potentially leading to referrals to counselors outside of school.^53^ This trend may also be associated with the scarcity of psychiatrists in Japan who can provide care in early adolescence^54,55^ and the limited accessibility of school counselors due to their part-time work.^56^ Among girls, individual-level prosociality was associated with help-seeking from others, potentially due to gender differences in online contact behaviors. Female youths are more likely to use helplines for mental health concerns,^57,58^ yet they also face heightened risks of online sexual offenses by strangers.^59^

### Strengths and Limitations

This study has some strengths owing to the S-ABC survey, which, developed in cooperation with school teachers, provided valuable insights into the school community. Moreover, this survey’s annual multiwave design enabled the examination of long-term community trends, including during the COVID-19 pandemic. Approximately 70% of schools participated in 3 of 4 waves, with approximately 50% of schools participating in all waves.

However, the study also has some limitations. The cross-sectional nature of the survey means that causality cannot be established, highlighting the need for further longitudinal and experimental studies to validate our findings. Furthermore, it was unclear whether survey items associated with help-seeking from physicians referred to mental health specialists (eg, psychiatrists) or general practitioners (eg, internal medicine physicians or pediatricians). Measurement of help-seeking behaviors was limited to a binary manner (present or absent), and future research is needed to examine the quality, frequency, or perceived effectiveness of help-seeking behaviors.

## Conclusions

This cross-sectional study of 21 845 students investigated the associations of both classroom-level and individual-level prosociality with help-seeking behaviors among adolescents, while accounting for individual characteristics, such as general psychopathology. No moderation effect of experiencing bullying was found in the association of prosociality with help-seeking from friends among senior high school students. Improving prosociality, both at the classroom and individual levels, may provide pathways to facilitate help-seeking behaviors, independent of the characteristics of individual adolescents. Further research is needed to elucidate the causal relationships and identify potential mediators for this dynamic.

## Notes

Supplement 1. **eMethods.** Study Design and Participants of the School Adolescent Behavior and Care (S-ABC) Survey
eReferences.
**eFigure.** Flowchart of Participant Selection **eTable 1.** Number (N) and Rate (%) of Missing Observations in Each Variable **eTable 2.** Values of Information Criteria for the Generalized Mixed-Effects Models **eTable 3.** Interaction Effects Between Community-Level and/or Individual-Level Prosociality and Grade (Junior High/Senior High School)/Gender (Boys/Girls) Subgroups on Help-Seeking Behaviors **eTable 4.** Values of Information Criteria for the Generalized Mixed-Effects Models Stratified by Grades **eTable 5.** Values of Information Criteria for the Generalized Mixed-Effects Models Stratified by Gender **eTable 6.** Interaction Effects Between Community-Level and/or Individual-Level Prosociality and Being Bullied Status on Help-Seeking Behaviors in Total Sample and Each Subgroup **eTable 7.** Sensitivity Analyses Stratified by Survey Year in the Generalized Mixed-Effects Models on Relationship Between Community-Level and Individual-Level Prosociality and Help-Seeking Behaviors

Supplement 2.
Data Sharing Statement
