# Supplementary material for: Classroom-Level and Individual-Level Prosociality and Help-Seeking Behaviors Among Adolescents
Source: JAMA Netw Open. 2025 May 15;8(5):e2510319. doi: 10.1001/jamanetworkopen.2025.10319 (PMC12082369; doi:10.1001/jamanetworkopen.2025.10319)
Supplement: Supplement 1. — eMethods. Study Design and Participants of the School Adolescent Behavior and Care (S-ABC) Survey eReferences. eFigure. Flowchart of Participant Selection eTable 1. Number (N) and Rate (%) of Missing Observations in Each Variable eTable 2. Values of Information Criteria for the Generalized Mixed-Effects Models eTable 3. Interaction Effects Between Community-Level and/or Individual-Level Prosociality and Grade (Junior High/Senior High School)/Gender (Boys/Girls) Subgroups on Help-Seeking Behaviors eTable 4. Values of Information Criteria for the Generalized Mixed-Effects Models Stratified by Grades eTable 5. Values of Information Criteria for the Generalized Mixed-Effects Models Stratified by Gender eTable 6. Interaction Effects Between Community-Level and/or Individual-Level Prosociality and Being Bullied Status on Help-Seeking Behaviors in Total Sample and Each Subgroup eTable 7. Sensitivity Analyses Stratified by Survey Year in the Generalized Mixed-Effects Models on Relationship Between Community-Level and Individual-Level Prosociality and Help-Seeking Behaviors [file jamanetwopen-e2510319-s001.pdf]

Supplementary Online Content

Morishima R, Usami S, Kanehara A, et al. Classroom-level and individual-level prosociality and help-seeking behaviors among adolescents. *JAMA Netw Open.* 2025;8(5):e2510319. doi:10.1001/jamanetworkopen.2025.10319

eMethods. Study Design and Participants of the School Adolescent Behavior and Care (S-ABC) Survey
eReferences.
eFigure. Flowchart of Participant Selection
eTable 1. Number (N) and Rate (%) of Missing Observations in Each Variable
eTable 2. Values of Information Criteria for the Generalized Mixed-Effects Models
eTable 3. Interaction Effects Between Community-Level and/or Individual-Level Prosociality and Grade (Junior High/Senior High School)/Gender (Boys/Girls) Subgroups on Help-Seeking Behaviors
eTable 4. Values of Information Criteria for the Generalized Mixed-Effects Models Stratified by Grades
eTable 5. Values of Information Criteria for the Generalized Mixed-Effects Models Stratified by Gender
eTable 6. Interaction Effects Between Community-Level and/or Individual-Level Prosociality and Being Bullied Status on Help-Seeking Behaviors in Total Sample and Each Subgroup
eTable 7. Sensitivity Analyses Stratified by Survey Year in the Generalized Mixed-Effects Models on Relationship Between Community-Level and Individual-Level Prosociality and Help-Seeking Behaviors

This supplementary material has been provided by the authors to give readers additional information about their work.

© 2025 Morishima R et al. *JAMA Network Open.*

eMethods. Study Design and Participants of the School Adolescent Behavior and Care (S-ABC) Survey

S-ABC is an annual multi-wave cross-sectional survey conducted in Japan. A detailed description of the survey can be also found in previous studies (e.g., Morishima et al., 2022; Morishima et al., 2023). The survey was distributed to private junior and senior high schools associated with the Association for Saitama Private Junior and Senior High Schools in Saitama Prefecture, which is home to a population of approximately 7.3 million people in Japan. This ongoing anonymous questionnaire survey commenced with, the first wave administered in 21 schools (nine junior and 12 senior high schools) from October to November 2020. Subsequent surveys followed: the second wave took place in 28 schools (11 junior and 17 senior high schools) from June to July 2021; the third wave in 26 schools (11 junior and 15 senior high schools) from June to July 2022; and the fourth wave in 25 schools (nine junior and 16 senior high schools) from June to July 2023. A total of 35 schools participated, with 17 schools (seven junior and 10 senior high schools) participated in all waves, seven schools (three junior and four senior high schools) involving in three times, and 11 schools (three junior and eight senior high schools) participating once.

Before administering the surveys, students and their parents were informed of the study's purpose and ethical considerations and that participation in the study was voluntary. On the day of implementing the surveys, teachers distributed the questionnaires to students and explained that the data collected would remain confidential and that there was no disadvantage to nonparticipation. The participants' willingness to participate was verified by obtaining their written informed consent. This study was approved by the Ethics Committee of the Faculty of the Medicine at the University of Tokyo [Approval No. 2019271NI-(5)], and Ethics Committee of Teikyo Heisei University [Approval No. 2024-013 and 2024-014]. This study was performed in accordance with the ethical standards of the 1964 Declaration of Helsinki and its later amendments.

© 2025 Morishima R et al. *JAMA Network Open.*

eReferences.

Morishima, R., Kanehara, A., Aizawa, T., Okada, N., Usui, K., Noguchi, H., & Kasai, K. (2023). Long-term trends and sociodemographic inequalities of emotional/behavioral problems and poor help-seeking in adolescents during the COVID-19 pandemic. Journal of Adolescent Health. https://doi.org/S1054-139X(23)00498-6 [Advance online publication]. https://doi.org/10.1016/j.jadohealth.2023.09.015.

Morishima, R., Koike, H., Kanehara, A., Usui, K., Okada, N., Ando, S., & Kasai, K. (2022). Implementation of online classes during national school closure due to COVID-19 and mental health symptoms of adolescents: A cross-sectional survey of 5000 students. PCN Reports: Psychiatry and Clinical Neurosciences, 1(2), e17. https://doi.org/10.1002/pcn5.17.

© 2025 Morishima R et al. *JAMA Network Open.*

eFigure. Flowchart of Participant Selection

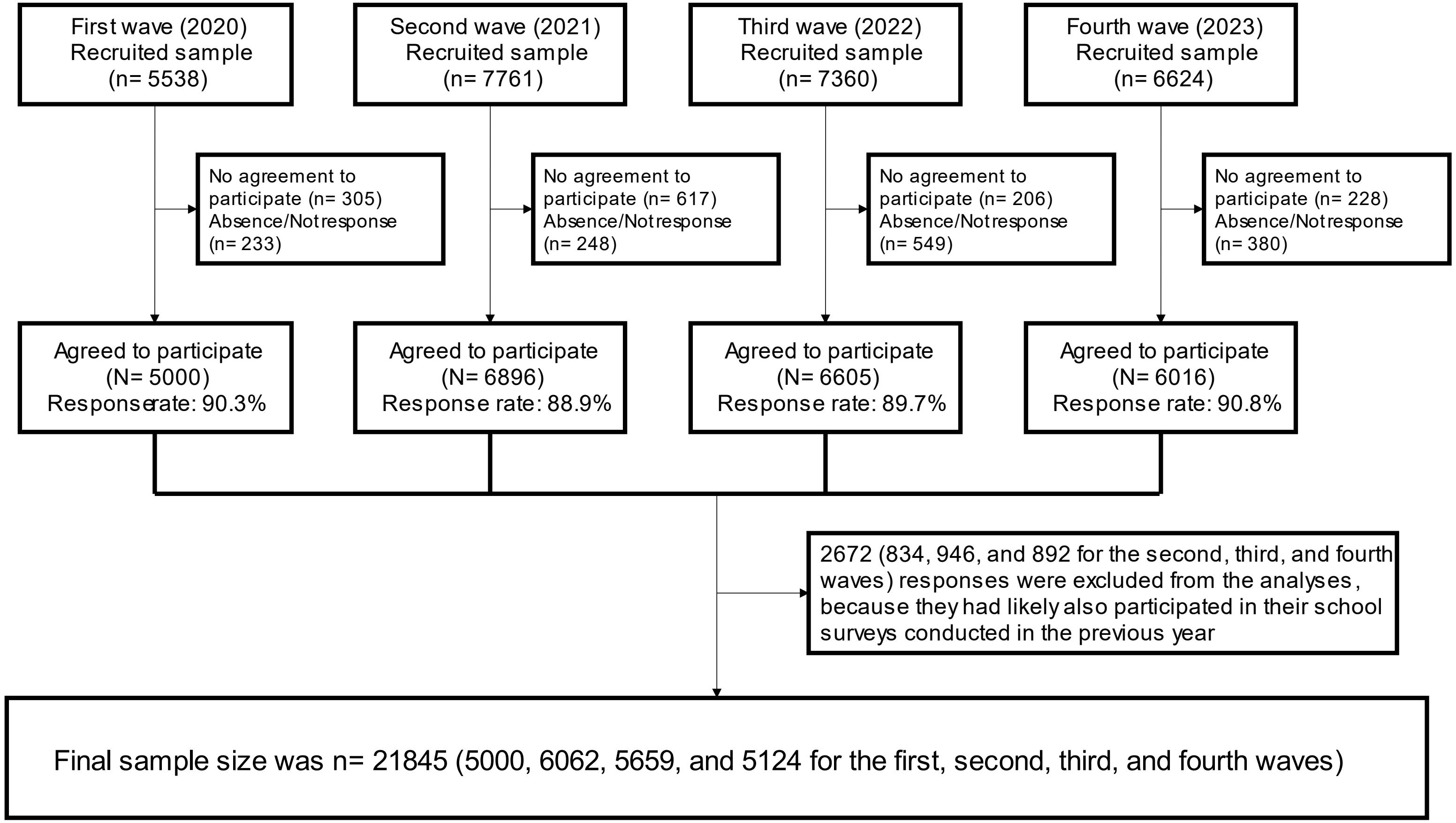

© 2025 Morishima R et al. *JAMA Network Open.*

eTable 1. Number (N) and Rate (%) of Missing Observations in Each Variable

	No. (%) of missing observations									
	Total		2020		2021		2022		2023	
Grades	0	(0.0)	0	(0.0)	0	(0.0)	0	(0.0)	0	(0.0)
Gender	155	(0.7)	23	(0.5)	42	(0.7)	56	(1.0)	34	(0.7)
General psychopathology	858	(4.1)	211	(4.4)	230	(3.9)	149	(2.7)	268	(5.5)
Being bullied	855	(4.1)	210	(4.4)	228	(3.9)	144	(2.6)	268	(5.5)
Prosociality	833	(4.0)	202	(4.2)	230	(3.9)	0	(0.0)	257	(5.3)
Poor help-seeking	0	(0.0)	0	(0.0)	0	(0.0)	0	(0.0)	0	(0.0)
Help-seeking from…										
Friends	0	(0.0)	0	(0.0)	0	(0.0)	0	(0.0)	0	(0.0)
Family members	0	(0.0)	0	(0.0)	0	(0.0)	0	(0.0)	0	(0.0)
Homeroom teacher	0	(0.0)	0	(0.0)	0	(0.0)	0	(0.0)	0	(0.0)
School nurses	0	(0.0)	0	(0.0)	0	(0.0)	0	(0.0)	0	(0.0)
School counselors	0	(0.0)	0	(0.0)	0	(0.0)	0	(0.0)	0	(0.0)
Counselors outside of school	0	(0.0)	0	(0.0)	0	(0.0)	0	(0.0)	0	(0.0)
Physicians	0	(0.0)	0	(0.0)	0	(0.0)	0	(0.0)	0	(0.0)
Others	0	(0.0)	0	(0.0)	0	(0.0)	0	(0.0)	0	(0.0)

© 2025 Morishima R et al. *JAMA Network Open.*

eTable 2. Values of Information Criteria for the Generalized Mixed-Effects Models

			Model 1	Model 2	Model 3	Model 4
Poor help-seeking		AIC	19714.7	**19713.9**	19718.5	19719.8
		BIC	**19770.7**	19785.8	19790.4	19815.7
Help-seeking from…	Friends	AIC	**24294.6**	24298.0	24297.3	24302.9
		BIC	**24350.6**	24369.9	24369.3	24398.8
	Family members	AIC	**23105.1**	23107.4	23108.0	23111.8
		BIC	**23161.0**	23179.3	23179.9	23207.7
	Homeroom teacher	AIC	5540.0	5542.9	**5532.4**	5536.4
		BIC	**5595.9**	5614.8	5604.3	5632.3
	School nurses	AIC	2282.9	2286.7	**2276.6**	2282.8
		BIC	**2338.8**	2358.7	2348.5	2378.7
	School counselors	AIC	1713.2	1716.7	**1710.3**	1714.3
		BIC	**1769.2**	1788.6	1782.2	1810.2
	Counselors outside of school	AIC	1334.5	1334.4	1323.2	**1319.6**
		BIC	**1390.5**	1406.4	1395.2	1415.5
	Physicians	AIC	3018.9	**3017.6**	3019.4	3020.1
		BIC	**3074.8**	3089.5	3091.4	3116.0
	Others	AIC	3586.6	3590.2	**3574.2**	3579.6
		BIC	**3642.6**	3662.1	3646.2	3675.5

Bold represents the lowest mean AIC and BIC values in 20 models generated by multiple imputations. All models adjusting grade, gender, and general psychopathology. AIC, Akaike information criterion; BIC, Bayesian information criterion.

Model 1: random intercept; Model 2: random intercept and random slope of classroom-level effect of prosociality; Model 3: random intercept and random slope of individual-level effect of prosociality; Model 4: random intercept and random slope of classroom-level and individual-level effects of prosociality.

© 2025 Morishima R et al. *JAMA Network Open.*

eTable 3. Interaction Effects Between Community-Level and/or Individual-Level Prosociality and Grade (Junior High/Senior High School)/Gender (Boys/Girls) Subgroups on Help-Seeking Behaviors

			Significance level of interaction with grades (*P* value)		Significance level of interaction with gender (*P* value)	
Poor help-seeking		Classroom-level Prosociality	0.379		0.544	
		Individual-level Prosociality	0.366		0.272	
Help-seeking from…	Friends	Classroom-level Prosociality	0.178		0.745	
		Individual-level Prosociality	**0.061**	**†**	0.546	
	Family members	Classroom-level Prosociality	0.423		**0.008**	**
		Individual-level Prosociality	0.265		0.860	
	Homeroom teacher	Classroom-level Prosociality	0.380		0.704	
		Individual-level Prosociality	0.106		0.534	
	School nurses	Classroom-level Prosociality	**0.078**	**†**	0.197	
		Individual-level Prosociality	0.500		0.561	
	School counselors	Classroom-level Prosociality	0.917		0.857	
		Individual-level Prosociality	0.398		0.599	
	Counselors outside of school	Classroom-level Prosociality	0.223		0.533	
		Individual-level Prosociality	**0.025**	*	0.797	
	Physicians	Classroom-level Prosociality	**0.025**	*	0.833	
		Individual-level Prosociality	0.353		0.141	
	Others	Classroom-level Prosociality	**0.044**	*	0.980	
		Individual-level Prosociality	0.108		**0.040**	*

†p < 0.10, *p < 0.05, **p < 0.01.

© 2025 Morishima R et al. *JAMA Network Open.*

eTable 4. Values of Information Criteria for the Generalized Mixed-Effects Models Stratified by Grades

			Model 1	Model 2	Model 3	Model 4
Junior high school						
Help-seeking from…	Friends	AIC	**3751.1**	3753.8	3753.4	3757.9
		BIC	**3788.3**	3803.3	3803.0	3826.1
	School nurses	AIC	402.8	406.6	**400.1**	406.3
		BIC	**439.9**	456.2	449.6	474.4
	Counselors outside of school	AIC	**245.6**	248.3	249.5	253.5
		BIC	**282.8**	297.8	299.0	321.6
	Physicians	AIC	**411.3**	414.7	415.3	420.9
		BIC	**448.5**	464.3	464.9	489.1
	Others	AIC	**829.6**	832.8	830.7	836.3
		BIC	**866.8**	882.4	880.3	904.4
Senior high school						
Help-seeking from…	Friends	AIC	**20541.7**	20545.2	20545.1	20551.1
		BIC	**20588.5**	20607.7	20607.6	20637.0
	School nurses	AIC	1885.5	1889.3	**1884.6**	1889.6
		BIC	**1932.4**	1951.8	1947.1	1975.5
	Counselors outside of school	AIC	1089.7	1091.7	1080.3	**1078.1**
		BIC	**1136.6**	1154.2	1142.8	1164.0
	Physicians	AIC	2601.0	**2599.8**	2600.5	2601.5
		BIC	**2647.9**	2662.3	2662.9	2687.4
	Others	AIC	2754.3	2757.9	**2743.0**	2747.9
		BIC	**2801.2**	2820.4	2805.5	2833.8

Bold represents the lowest mean AIC and BIC values in 20 models generated by multiple imputations. All models adjusting gender and general psychopathology. AIC, Akaike information criterion; BIC, Bayesian information criterion.

Model 1: random intercept; Model 2: random intercept and random slope of classroom-level effect of prosociality; Model 3: random intercept and random slope of individual-level effect of prosociality; Model 4: random intercept and random slope of classroom-level and individual-level effects of prosociality.

© 2025 Morishima R et al. *JAMA Network Open.*

eTable 5. Values of Information Criteria for the Generalized Mixed-Effects Models Stratified by Gender

			Model 1	Model 2	Model 3	Model 4
Boys						
Help-seeking from…	Family members	AIC	**10343.2**	10344.9	10346.5	10349.5
		BIC	**10387.4**	10403.8	10405.5	10430.6
	Others	AIC	1480.8	1484.8	**1459.4**	1463.6
		BIC	1525.0	1543.8	**1518.4**	1544.6
Girls						
Help-seeking from…	Family members	AIC	**12584.0**	12585.0	12588.0	12591.0
		BIC	**12627.3**	12642.7	12645.7	12670.3
	Others	AIC	2041.8	2042.2	**2039.3**	2043.1
		BIC	**2085.1**	2099.9	2097.0	2122.4

Bold represents the lowest mean AIC and BIC values in 20 models generated by multiple imputations. All models adjusting grades and general psychopathology. AIC, Akaike information criterion; BIC, Bayesian information criterion.

Model 1: random intercept; Model 2: random intercept and random slope of classroom-level effect of prosociality; Model 3: random intercept and random slope of individual-level effect of prosociality; Model 4: random intercept and random slope of classroom-level and individual-level effects of prosociality.

© 2025 Morishima R et al. *JAMA Network Open.*

eTable 6. Interaction Effects Between Community-Level and/or Individual-Level Prosociality and Being Bullied Status on Help-Seeking Behaviors in Total Sample and Each Subgroup

			Significance level of interaction with being bullied (p-value)				
			Total sample	Junior high school students	Senior high school students	Boys	Girls
Poor help-seeking		Classroom-level Prosociality	0.171				
		Individual-level Prosociality	0.432				
Help-seeking from…	Friends	Classroom-level Prosociality	0.319	0.920	0.280		
		Individual-level Prosociality	0.564	**0.047** *	0.149		
	Family members	Classroom-level Prosociality	**0.003** **			**0.024** *****	0.176
		Individual-level Prosociality	0.535			0.729	0.376
	Homeroom teacher	Classroom-level Prosociality	0.130				
		Individual-level Prosociality	**0.006** **				
	School nurses	Classroom-level Prosociality	0.879	0.794	0.855		
		Individual-level Prosociality	0.822	0.557	0.890		
	School counselors	Classroom-level Prosociality	0.241				
		Individual-level Prosociality	0.187				
	Counselors outside of school	Classroom-level Prosociality	0.243	0.766	0.174		
		Individual-level Prosociality	0.436	0.950	0.506		
	Physicians	Classroom-level Prosociality	0.334	0.887	0.355		
		Individual-level Prosociality	0.204	0.719	0.124		
	Others	Classroom-level Prosociality	0.889	0.302	0.708	0.537	0.492
		Individual-level Prosociality	0.581	0.631	0.508	0.227	0.807

*p < 0.05, **p < 0.01.

© 2025 Morishima R et al. *JAMA Network Open.*

eTable 7. Sensitivity Analyses Stratified by Survey Year in the Generalized Mixed-Effects Models on Relationship Between Community-Level and Individual-Level Prosociality and Help-Seeking Behaviors

			2020		2021		2022		2023	
			OR	*P* value	OR	*P* value	OR	*P* value	OR	*P* value
Total sample[a]										
Poor help-seeking		Classroom-level Prosociality	0.981 [0.845 to 1.137]	0.796	0.887 [0.781 to 1.007]	0.064	0.890 [0.782 to 1.014]	0.080	**0.795 [0.669 to 0.944]**	**0.009**
		Individual-level Prosociality	**0.927 [0.893 to 0.962]**	**<0.001**	0.973 [0.942 to 1.004]	0.084	0.981 [0.949 to 1.014]	0.258	0.972 [0.939 to 1.006]	0.108
Junior high school students[b]										
Help-seeking from…	Physicians	Classroom-level Prosociality	**7.164 [2.182 to 23.515]**	**0.001**	1.185 [0.159 to 8.862]	0.868	2.347 [0.722 to 7.630]	0.156	0.652 [0.085 to 4.989]	0.680
		Individual-level Prosociality	0.960 [0.711 to 1.297]	0.792	1.078 [0.762 to 1.524]	0.671	0.988 [0.745 to 1.310]	0.933	1.396 [0.976 to 1.997]	0.068
Senior high school students[b]										
Help-seeking from…	Friends	Classroom-level Prosociality	**1.244 [1.070 to 1.445]**	**0.004**	**1.283 [1.138 to 1.446]**	**<0.001**	**1.231 [1.070 to 1.415]**	**0.004**	**1.278 [1.080 to 1.513]**	**0.004**
		Individual-level Prosociality	**1.185 [1.144 to 1.227]**	**<0.001**	**1.142 [1.106 to 1.180]**	**<0.001**	**1.153 [1.115 to 1.191]**	**<0.001**	**1.147 [1.108 to 1.189]**	**<0.001**

OR, odds ratios; CI, confidence interval.

[a]Model adjusting grade, gender, and general psychopathology

[b]Model adjusting gender, and general psychopathology.

© 2025 Morishima R et al. *JAMA Network Open.*
