# Supplementary material for: Classroom-Level and Individual-Level Prosociality and Help-Seeking Behaviors Among Adolescents
Source: JAMA Netw Open. 2025 May 15;8(5):e2510319. doi: 10.1001/jamanetworkopen.2025.10319 (PMC12082369; doi:10.1001/jamanetworkopen.2025.10319)
Supplement: Supplement 2. — Data Sharing Statement [file jamanetwopen-e2510319-s002.pdf]

Data Sharing Statement

Morishima. Classroom-Level and Individual-Level Prosociality and Help-Seeking Behaviors Among Adolescents. *JAMA Netw Open*. Published May 15, 2025. doi:10.1001/jamanetworkopen.2025.10319

Data
Data available: No

Additional Information
Explanation for why data not available: The data used in this study are not available for open access because of the provisions of the ethics committee and participant consent agreements. Requests to access the data can be directed to the corresponding author and the Ethics Committee of the Faculty of Medicine at the University of Tokyo.
